# Biomimetic Natural Biomaterial Nanocomposite Scaffolds: A Rising Prospect for Bone Replacement

**DOI:** 10.3390/ijms252413467

**Published:** 2024-12-16

**Authors:** Maja A. Zaczek-Moczydłowska, Kamil Joszko, Mahboubeh Kavoosi, Aleksandra Markowska, Wirginia Likus, Saeid Ghavami, Marek J. Łos

**Affiliations:** 1 Biotechnology Center, The Silesian University of Technology, 44-100 Gliwice, Poland; 2 Department of Biomechatronics, Faculty of Biomedical Engineering, The Silesian University of Technology, 41-800 Zabrze, Poland; 3 Faculty of Medicine, Medical University of Warsaw, 02-091 Warsaw, Poland; 4 Department of Anatomy, Faculty of Health Sciences, Medical University of Silesia in Katowice, 40-752 Katowice, Poland; 5 Department of Human Anatomy and Cell Science, Rady Faculty of Health Sciences, Max Rady College of Medicine, University of Manitoba, Winnipeg, MB R3E 0T6, Canada; 6 Faculty of Medicine, Academy of Silesia, 40-555 Katowice, Poland; 7 Paul Albrechtsen Research Institute, CancerCare Manitoba, Winnipeg, MB R3E 0V9, Canada

**Keywords:** BNBM nanocomposites, biopolymers, nanoparticles, bone replacement, biomaterials

## Abstract

Biomimetic natural biomaterial (BNBM) nanocomposite scaffolds for bone replacement can reduce the rate of implant failure and the associated risks of post-surgical complications for patients. Traditional bone implants, like allografts, and autografts, have limitations, such as donor site morbidity and potential patient inflammation. Over two million bone transplant procedures are performed yearly, and success varies depending on the material used. This emphasizes the importance of developing new biomaterials for bone replacement. Innovative BNBM nanocomposites for modern bone fabrication can promote the colonization of the desired cellular components and provide the necessary mechanical properties. Recent studies have highlighted the advantages of BNBM nanocomposites for bone replacement; therefore, this review focuses on the application of cellulose, chitosan, alginates, collagen, hyaluronic acid, and synthetic polymers enhanced with nanoparticles for the fabrication of nanocomposite scaffolds used in bone regeneration and replacement. This work outlines the most up-to-date overview and perspectives of selected promising BNBM nanocomposites for bone replacement that could be used for scaffold fabrication and replace other biomorphic materials such as metallics, ceramics, and synthetic polymers in the future. In summary, the concluding remarks highlight the advantages and disadvantages of BNBM nanocomposites, prospects, and future directions for bone tissue regeneration and replacement.

## 1. Introduction

In the past thirty years, there has been significant advancement in the development of bone substitute materials that can replace natural bone grafts. This progress ranges from the use of metal alloys, ceramics, and polymers for bone implant engineering to the utilization of advanced nanomaterials for crafting three-dimensional (3D) scaffolds to treat bone defects and enhance bone tissue regeneration [1,2,3,4].

Thus far, ‘the gold standard’ grafts, autografts, and allografts have been primarily considered for transplantation, with two million bone transplants performed each year worldwide [5,6,7]. Allograft transplantation accounts for 57% of the grafts available on the market [8]. The main drawback of allograft implantations is the potential inflammation due to an immune response to the implant [7]. Xenografts are another type of graft derived from animal organs/tissue that can serve as a substitute for a human bone. Xenografts are rarely used because of the risk of transmitting pathogens into the host organism and potentially spreading from person to person, creating public health concerns [8].

**Figure 1:**
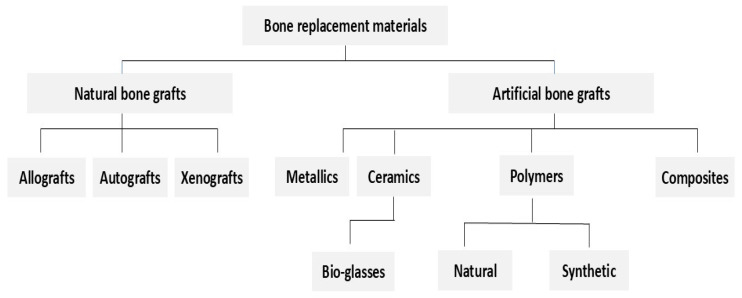
Schematic summary of bone replacement materials. Presented bone replacement materials are classified into two main groups, including natural and artificial bone grafts, which are further sub-classified.

An alternative to natural bone grafts is an artificial implant made from biomaterials (bioinert, bioactive, and biocompatible) with good mechanical properties and the ability to respond to cellular signals for regeneration [9]. The earliest materials used to ensure the restoration of damaged or fractured bone were several metal alloys, such as chromium-nickel–molybdenum steels (e.g., 316L stainless steel) and titanium alloys with various elements such as niobium (i.e., Ti6Al7Nb) or vanadium (i.e., Ti6Al4V). The main advantage of this group is their very good mechanical properties, such as resistance to bending, stretching, and torsion. Common obstacles to using metallic implants are local tissue damage or inflammatory reactions. Bioceramics and bio-glasses, including aluminum oxide, zirconium oxide, calcium phosphates, and carbon, are another significant group of biomaterials used for bone replacement due to their positive interaction with human tissue. Artificially obtained bioceramics stand out for their good biocompatibility and osteoconductive properties. However, they degrade slowly and have poor mechanical properties [10,11,12]. Polymers are characterized by good biocompatibility, biodegradability, and ease of molding. Common drawbacks of using polymers as bone implants include poor compression strength and the possibility of an inflammatory response [7,10,11,12]. The different types of biomaterials commonly used for bone replacement are summarized in Figure 1.

A selected artificial biomaterial used to replace a bone, whether composed of ceramics, metal, or polymers, must be safe and meet biocompatibility criteria to prevent toxicity, irritation, inflammation, or an immune response. Guidelines for the biocompatibility, safety, and efficacy of biomaterials are available in established documents and standards, such as the International Organization for Standardization 10993, Good Laboratory Practice, and the Organization for Economic Co-operation and Development, issued by Global Regulatory Agencies appropriate for countries and regions. For example, the European Commission, Medicines Agency, Healthcare Products Regulatory Agency in Europe, and The Center for Devices and Radiologic Health in the United States help establish guidelines [13]. According to these guidelines, biomaterials used for bone replacement should meet the criteria for bioactivity, osteoconductivity, and osteoinductivity [14,15,16,17]. Additionally, having a specific composition or surface modifications that enable the ‘programming’ of cell fate (the differentiation and maintenance of the desired cellular phenotype) is a significant advantage [8,18,19].

BNBMs, specifically biopolymers, can mimic the structure and mechanics of bone for regeneration and reconstruction [20]. Hybrid nanocomposites consisting of BNBMs have shown great potential for developing bone implants and offer a unique opportunity to create scaffolds with the desired biological, physical, and structural properties [12]. For instance, they can be used for printing 3D tissue-mimicking scaffolds with adequate implant porosity, promoting the osteogenesis of cells, and possessing antibacterial properties to prevent post-surgical infections [11]. The challenge in selecting BNBM nanocomposites for bone replacement is finding materials that are biodegradable, bioactive, and mechanically strong.

This review is prompted by the increasing number of recent reports that emphasize the potential of these materials for bone replacement. Therefore, this study aims to summarize current trends and achievements in BNBM nanocomposites development for bone replacement and regeneration, both in vitro and in vivo. This review also includes a critical evaluation of selected BNBMs compared to conventional biomorphic biomaterials. Finally, based on the existing literature and research experience, the potential and future outlook of BNBM nanocomposites as bone replacement candidates are discussed and reviewed.

## 2. BNBM Nanocomposites for Bone Replacement

The functionalization of BNBMs with several nanoparticles is actively being explored to enhance the properties of the implant–bone interface and counteract potential bacterial infection while also increasing overall biocompatibility towards osteoblasts and osteocytes. This can be achieved by increasing surface wettability and porosity, and modifying the surface topology to obtain desired patterns that improve cell–implant interactions and maintain or favor certain cellular phenotypes.

**Figure 2:**
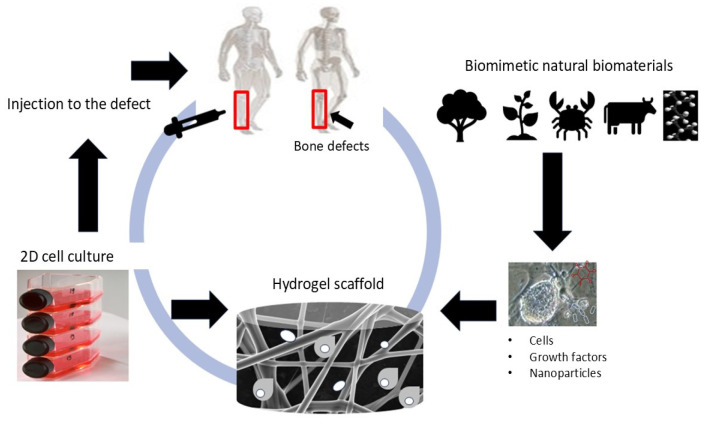
Schematic of the development of BNBM nanocomposites for bone replacement.

This section provides an overview of advancements in the development of nanocomposites based on selected BNBMs, including cellulose, alginates, chitosan, hyaluronic acid (HA), collagen, and synthetic biopolymers. A schematic of the development of BNBM nanocomposites for the treatment of bone defects is depicted in Figure 2.

### 2.1. Cellulose

Cellulose is a biodegradable biomaterial that is stable under acidic conditions and characterized by a high tensile strength [21,22]. A simple conjugation procedure with other biopolymers and/or nanoparticles makes cellulose a potentially valuable material for bone grafting (Table 1).

For example, nanocomposites consisting of hydroxyapatite (HAp)/cellulose or cellulose/polyacrylamide/titanium dioxide (TiO_2_) improve the roughness, biological, and mechanical properties of the cellulose scaffold necessary for implant fabrication [23,24]. The prospective approach to improving bacterial cellulose (BC) properties is to incorporate nanoparticles of magnesium oxide (MgONPs), silver (AgNPs), or bioactive glass (BGNPs). Scaffolds produced this way can enhance processes involved in direct bone apposition into biomaterials, including proliferation, differentiation, adhesion, and viability of the cells (Table 1).

**Table 1:** Cellulose-based nanocomposite scaffolds, prospects, and limitations.

Nanoparticles	Scaffold Composition	Prospects/Limitations	Ref.
AgNPs ^1^ HApNPs ^2^	AgNPs ^1^/HApNPs ^2^ on PLA ^3^/CA ^4^ or PCL ^5^	Improved cell viability; antibacterial activity; desirable degradation profile. Future studies should include in vivo testing.	[25]
MgONPs ^6^	MgONPs ^6^ and BC ^7^ nanofibers	Increased mechanical strength and porosity; enhanced ALP ^8^ and OCN ^9^ gene expression rates; increased osteoblast proliferation and adhesion; greater calcium deposition. Osteogenic effects of MgONP ^6^-BC ^7^ need further investigation in vivo.	[26]
CNCs ^10^	CNCs ^10^ combined with platelet lysate	Upregulation of RUNX2 ^11^, BMP-2 ^12^, and COL1A1 ^13^; increased ALP ^8^; proangiogenic effects via promoting chemotaxis of endothelial cells; enhancing the expression of endothelial markers. In vivo studies are necessary to validate the results.	[27]
BGNPs ^14^	BC ^7^/SF ^15^ with BGNPs ^14^	Increased expression of RUNX2 ^11^, ALP ^8^, and OCN ^9^ genes; increased cell adhesion, viability, and differentiation; improved storage modulus and compressive strength of the scaffold. In vivo animal studies are necessary to investigate the applicability of the SF ^15^/BC ^7^ scaffolds.	[28]

^1^ Silver nanoparticles (AgNPs); ^2^ hydroxyapatite nanoparticles (HApNPs); ^3^ poly (lactic acid) (PLA); ^4^ cellulose acetate (CA); ^5^ polycaprolactone (PCL) polymers; ^6^ magnesium oxide nanoparticles (MgONPs); ^7^ bacterial cellulose (BC); ^8^ alkaline phosphatase (ALP); ^9^ osteocalcin (OCN); ^10^ mineralized cellulose nanocrystals (CNCs); ^11^ runt-related transcription factor 2 (RUNX2); ^12^ bone morphogenetic protein-2 (BMP-2); ^13^ collagen type 1 alpha 1 (COLIA1); ^14^ bioactive glass nanoparticles (BGNPs); ^15^ silk fibroin (SF).

The fabrication of BC nanocomposites consisting of MgONPs and BGNPs can enhance the osteointegration process, which is important for the long-term clinical success of bone implants. The reported nanocomposites promote the secretion of osteogenic markers such as ALP, OCN, and RUNX2, which are involved in osteogenesis (the migration of osteogenic cells along the implant to form a matrix separating the old and new bones) in comparison to BC scaffolds without nanoparticles. Additionally, MgONPs and BGNPs significantly improved the mechanical strength and porosity of the BC hydrogels [26,28]. These two studies provided successful in vitro trials that are necessary for the development of bone implants that could be introduced for clinical bone defect treatment. However, further exploration in animal models is still necessary to confirm the real applicability of the designed scaffolds. An engineered nanocomposite scaffold consisting of SF/BC/BGNPs could potentially be used as a bioink in 3D printing to develop a cell matrix for bone restoration. However, to understand the interaction mechanism of the extracellular matrix (ECM) with cells in fabricated scaffolds, protein expression of bone cells in different periods of stem cell differentiation should be further investigated. It would also be important to test SF/BC scaffolds with other natural or synthetic polymers for nanocomposite improvement and to compare potential discrepancies in this study [28].

Another type of cellulose, known as CNCs, exhibits favorable biocompatibility and good mechanical properties for bone regeneration. For instance, incorporating CNCs into scaffolds can enhance their compressive strength, wettability (resulting in better adhesion), and osteoblast differentiation [29,30]. Fabricated nanocomposite scaffolds consisting of CNCs and platelet lysate up-regulated the expression of osteogenic markers RUNX2, BMP-2, and COL1A1. Furthermore, CNC-incorporated scaffolds promote the chemotaxis of endothelial cells and increase the expression of an endothelial marker, CD31, which is important for leukocyte transmigration across the endothelium. This scaffold was also characterized by high microporosity, elasticity, and injectability [27]. Given these promising properties of CNCs as biomaterials, further research is needed to explore their potential for treating bone defects in vivo.

### 2.2. Alginates

Thus far, several nanoparticles have been reported to enhance the properties of fabricated alginate scaffolds, demonstrating advancements in mechanical performance, cell proliferation, viability, and attachment when tested under in vitro conditions (Table 2).

A nanocomposite, consisting of gelatin–alginate scaffolds with NC nanoparticles, has been reported to promote the critical process of osteogenic differentiation, enabling osteoblast and bone healing. This promising scaffold can scavenge free radicals, which are involved in increasing oxidative stress, a known factor that causes bone healing pathologies (e.g., inflammation or improper wound healing) and, for example, enhances osteoclast behavior to reduce bone mineral density [32] (Table 2).

Another interesting approach involves the synergistic cooperation of 3D-printed PDA/SiO_2_-CaO nanoparticle complexes incorporated into ADA-GEL scaffolds. This resulted in a more stable construct with controllable biodegradation behavior, creating a better platform for the adhesion and proliferation of preosteoclast cells involved in the replacement process of damaged bone cells. In this case, an in-depth exploration of cellular responses, vascularization strategies, and immune reactions in vivo would provide new insights into the bone healing process. Investigations into the scalability and cost-effectiveness of the fabrication process of the nanocomposite for clinical translation would also be important for this approach [34].

**Figure 3:**
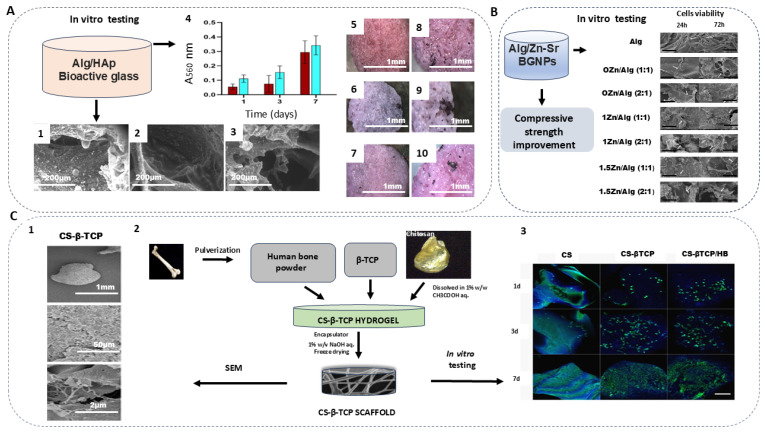
In vitro results of reviewed alginate- and chitosan-based nanocomposites for bone regeneration. (**A**) Nanocomposite consisting of alginate/HAp/BGs (**1**–**3**). Nanocomposite morphology obtained using eSEM: (**1**) Ctrl-sc; (**2**) BG6-sc; (**3**) BG12-sc; (**4**) MTT assay of MG63 viability on Ctrl-sc (blue) and BG6-sc (red) scaffolds. (**5**–**10**) Top views obtained using stereoscope: (**5**,**8**) Ctrl-sc; (**6**,**9**) BG6-sc; (**7**,**10**) BG12-sc; black spots are cells which metabolized the MTT dye; BG6-sc represents a higher level of cell colonization [36]. (**B**) Compressive strength improvement and cell viability of nanocomposite consisting of alginate/Zn-Sr-BGNPs; Scale bar: 50 µm (operate at 15.0 kV, 500×) [33]. (**C**) Nanocomposite consisting of chitosan (CS), β-tricalcium phosphate (β-TCP), and human bone powder: (**1**) TEM images of CS-β-TCP; (**2**) schematic of the development of CS-β-TCP from chitosan, human bone powder, and β-TCP; (**3**) cell viability of developed nanocomposite. Scale bar—200 µm [37].

Two other in vitro studies reported possibilities to trigger the biological properties of alginate scaffolds using BGNPs for bone regeneration. The developed porous and biocompatible nanocomposite consisting of alginate-Sr/Mg and BGs enhanced cell adhesion, colonization, and proliferation (Figure 3A) [36]. Another, fabricated alginate nanocomposite consisting of Zn-Sr-BGNPs, improved the compressive strength and enhanced the cell proliferation of alginate nanocomposites (Figure 3B).

### 2.3. Chitosan

Several in vitro studies highlight the benefits arising from using chitosan to fabricate BNBM nanocomposite scaffolds (Table 3).

For instance, a developed granulate composition containing *β*-TCP, pulverized human bone, and chitosan has been proven to be non-toxic, activate ALP activity (a marker for bone tissue growth), and enhance cell growth on its surface (Figure 3C). Additionally, the suitability of using thermal sterilization for this nanocomposite without losing properties is a significant advantage of this scaffold compared to the other biomaterials. Therefore, this biomaterial may be applied for bone regeneration [37]. Another research group investigated deacetylation (DDA) levels using a composite that contains injectable chitosan and a calcium phosphate-based composite with a potential application for bone regeneration [38]. The data (50–70% DDA) suggested that chitosan enhanced the natural bone regeneration process [38]. The incorporation of MgO nanoparticles into a phosphocreatine-functionalized chitosan (CSMP) water solution to form an injectable hydrogel with improved compressive strength, swelling resistance properties, and the controlled release of magnesium ions is also a prospective nanocomposite. CSMP-MgO injectable hydrogels promoted calcium phosphate deposition, cell proliferation, and osteogenic differentiation in vitro. This nanocomposite successfully promotes the mineralization of bones and up-regulates the expression of osteogenic bone genes such as sialoprotein, osteopontin, and osterix. Thus, the novel mechanisms of osteogenesis and angiogenesis of the hydrogel should be explored further along with the mechanical stability during in vitro degradation [39] (Table 3).

**Table 3:** Chitosan-based scaffolds, prospects, and limitations.

Nanoparticles	Scaffold Composition	Prospects/Limitations	Ref.
MgO ^1^	MgO ^1^ incorporated into CSMP ^2^	Promoting calcium and tetracalcium phosphates deposition promotes preosteoblast cell proliferation; lack of cytotoxicity. Lack of complete degradation was noted.	[39]
Chitosan-ciprofloxacin -chitosan—BMP-2 ^3^ nanoparticles	Ciprofloxacin, BMP-2 ^3^, and chitosan nanofibers	Rapid antibacterial prevention of graft infection; programmed release of BMP-2 ^3^; osteogenic differentiation of MSCs ^4^. Future studies should involve in vivo testing.	[40]
SrO_2_ ^5^ SPION ^6^ fMWCNTs ^7^	Chitosan nanonets on PU ^8^ biomembranes with SrO_2_ ^5^, SPION ^6^, fMWCNTs ^7^	Upregulation of ALP ^9^, ARS ^10^, COL-1 ^11^, RUNX2 ^12^, and SPP-1 ^13^; high electrical conductivity; antibacterial conductivity; water retention; biomineralization and load-bearing capability; improved cell survival; enhanced osteogenic differentiation. Future studies should involve in vivo testing.	[41]
MgO ^1^	PVA ^14^/chitosan-MgO ^1^-BPNS ^15^	Improvement of biomineralization; osteogenesis; antibacterial effect.	[42]
TiO_2_ ^16^	Chitosan sponge coated with TiO_2_ ^16^	Improved sponge robustness; biomineralization; bone regeneration capability via DMP1 ^17^; OCN ^18^ gene upregulation. The scaffold should be tested in vivo.	[43]
MHC NPs ^19^	Metformin, human serum albumin, and MHC NPs ^19^	Upregulation of OPG ^20^ and OCN ^18^ expression; stable metformin release effect. In vivo studies should be carried out.	[44]
Orsellinic acid and chitosan	Orsellinic acid, chitosan incorporated into the gelatin and nHAp ^21^	Enhanced differentiation of mMSCs ^4^ towards osteoblasts via cell adhesion-mediated signaling. In vivo testing should be performed.	[45]

^1^ Magnesium oxide (MgO); ^2^ phosphocreatine-functionalized chitosan (CSMP); ^3^ bone morphogenetic protein-2 (BMP-2); ^4^ mesenchymal stem cells (MSCs); ^5^ strontium peroxide (SrO_2_); ^6^ superparamagnetic iron oxide (SPION); ^7^ functionalized multiwall carbon nanotubes (fMWCNTs); ^8^ fibro-porous polyurethane (PU); ^9^ alkaline phosphatases (ALPs); ^10^ an autonomously replicating sequence (ARS); ^11^ collagen type I alpha 1 (COL-1); ^12^ runt-related transcription factor 2 (RUNX2); ^13^ secreted phosphoprotein 1 (SPP-1); ^14^ poly (vinyl alcohol) (PVA); ^15^ black phosphorus nanosheet (BPNS); ^16^ titanium dioxide (TiO_2_); ^17^ dentin matrix protein 1 (DMP1); ^18^ osteocalcin (OCN); ^19^ major histocompatibility complex nanoparticles (MHC NPs); ^20^ osteoprotegerin (OPG); ^21^ nanohydroxyapatite (nHAp).

A prospective in vitro study highlighted chitosan nanonets on PU bio-membranes comprising fMWCNTs, well-dispersed SPIONs, and SrO_2_ nanoparticles as a potential scaffold. The scaffold exhibited high electrical conductivity, biomineralization, water retention, load-bearing capability, and antibacterial activity; up-regulated the expressions of osteoinductive markers such as ALP, COL-I, and RUNX2; and secreted SPP–I (Table 3) [41].

Another in vitro study analyzed chitosan hybridized with TiO_2_ nanoparticles, which improved sponge robustness, biomineralization, and bone regeneration capability by increasing DMP1 and OCN gene expressions for efficient bone healing [43] (Table 3).

### 2.4. Hyaluronic Acid

The applicability of HA for bone regeneration relies on its biocompatibility, biodegradability, interaction with cells, and growth factors [45] (Table 4).

A study used encapsulated SLNs in a HA-PCLA hydrogel scaffold to form an anti-inflammatory microenvironment by decreasing M1 polarization and elevating M2 polarization. Enhanced BMP-2 and vascular endothelial growth factor (VEGF) paracrine signaling in M2 macrophages triggered osteoblast differentiation and vascularization in bone marrow stromal cells (BMSCs), with the synergistic enhancement of bone regeneration [47]. However, further investigation of the quercetin mechanisms affecting macrophage polarization and other tests performed in vivo for clinical application are still needed [47].

Another study analyzed nanocomposites consisting of MBG. Ipriflavone incorporated on HA proved to be a suitable vehicle for injecting MBGNs leading to the in situ formation of a highly hydrated gel. However, the moderate effect on osteogenesis presented by ipriflavone-loaded nanoparticles compared to unloaded particles could not be considered significant under the conditions of the study. Future research incorporating angiogenic ions such as cobalt (Co^2+^) and copper (Cu^2+^) could provide better results due to synergistic effects between ipriflavone and these ions [48].

The encapsulation of BGNPs with ipriflavone or MNCl) in a HA hydrogel to reduce the long-term inflammatory response that impairs the bone regeneration process is another interesting approach [49]. This in vitro study developed a photo-crosslinkable methacrylic anhydride (MHA) gel functionalized by MBGNs loaded with MNCl, which inhibited the expression of pro-inflammatory interleukin-6 (IL-6) and tumor necrosis factor-alpha (TNF-α); promoted the osteogenic gene expression of ALP, Runx2, and OPN; and inhibited the proliferation of *Streptococcus* mutants [49].

In a bone regeneration process, HA provides a similar extracellular microenvironment by interacting with cellular surface receptors such as CD44, CD168, and lymphatic vessel endothelial hyaluronan receptor 1, which activates intracellular pathways involved in cell adhesion, proliferation, differentiation, and degradation. Moreover, HA’s acidic functional groups act as binding domains for calcium ions, enhancing in situ biomineralization [50]. The development of biocompatible collagen/chitosan/lysine-modified HA-based (ColChHAmod) hydrogels has been reported as a structurally stable and improved form of hydrogel. It has been noted that by adjusting the content of HA and the concentration of genipin, hydrogels can achieve properties appropriate for bone tissue regeneration [51].

### 2.5. Collagen

**Figure 4:**
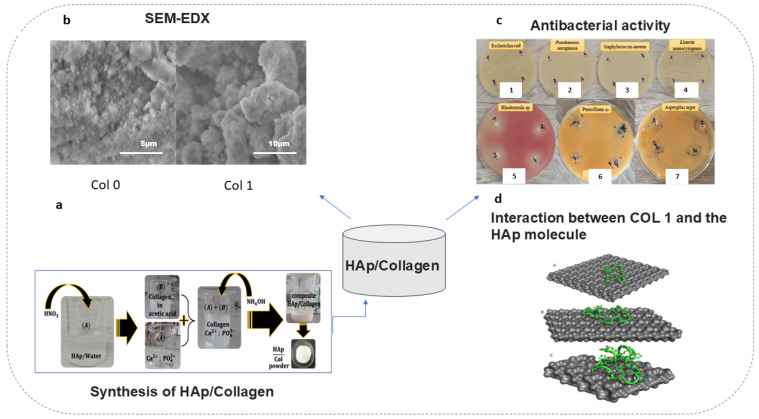
Nanocomposite consisting of HAp and collagen designed using a cheminformatics approach. (**a**) Synthesis of HAp/collagen (Col) nanocomposite; (**b**) identification using SEM-EDX analysis of composites (Col 0 and Col 1); (**c**) antibacterial and antifungal effects of samples on several bacterial and fungal strains: (1) *Escherichia coli*; (2) *Pseudomonas aeruginosa*; (3) *Staphylococcus aureus*; (4) *Listeria monocytogenes*; (5) *Rhodotorula* sp.; (6) *Penicillium* sp.; (7) *Aspergilus niger* (**d**) ADVina-predicted interaction between Col 1 and the Hap molecule for the Col 1, Col 2, and Col 3 complexes. The protein molecule is presented in both cartoon and ball-and-stick representations, with colors corresponding to its atomic composition. For clarity purposes, all hydrogen atoms are omitted from the visualization [59].

In the area of bone regeneration, collagen is used as a scaffold to provide a surface for bone superstructure and gene/cell matrices due to collagen’s high biodegradability, bioactivity, and relative ease of attachment to several types of cells [52,53,54,55,56]. Recently, a prospective recombinant monomeric polypeptide based on the human type I collagen alpha 1 chain with sequence modifications (RCPhC1) has been used to produce synthetic bone grafts with several successes [57]. Pre-clinical studies showed that RCPhC1-based bone grafts with highly porous granules and an optimized biodegradation rate enhance healing properties [57]. Other innovative methods have been discussed and can improve mechanical strength and enhance the fast biodegradability rate of collagen. Nanocomposites consisting of nanosilica-modified collagen (nSC) with refined mechanical properties increased hardness and reduced modulus. Similarly, the composites also provided a microenvironment that promotes the proliferation and osteogenic differentiation of mesenchymal stem cells (MSCs). Additionally, the negatively charged silanol groups of nanosilica provide strong interactions of the scaffold with mineral ions, leading to increased calcification [58]. A new cheminformatics method for developing HAp/collagen nanocomposites for use in orthopedics has recently been highlighted (Figure 4). However, in silico predictions of correlation (using bioinformatics tools) suitable for the antibacterial activity of this nanocomposite could be a possibility. Moreover, antibacterial activity was confirmed in laboratory conditions against several species of bacteria (Figure 4c). However, the biocompatibility and bioactivity of nanocomposites in vivo and the potential applications and functionalization of these innovative materials need to be explored further [59].

Reinforcing collagen with strontium–graphene oxide (Sr-GO) nanocomposites is associated with the long-term release of Sr ions, high water retention rates, and excellent mechanical properties. The modified scaffold enhances cell adhesion and osteogenic differentiation. The synergistic effect of GO and Sr on the activation of the MAPK signaling pathway increases the secretion of angiogenic and osteogenic proteins [60] (Table 5).

Recently, fabricated ciprofloxacin- and dexamethasone-loaded strontium- and iron-substituted HAp nanomaterials precipitated in collagen have demonstrated excellent antibacterial activity. They also showed an increased gene expression of osteogenic markers and the degradation of the matrix over time by controlling the release of ions into the body without causing acute inflammation [63]. Collagen is a promising biomaterial, and several bone implants based on recombinant collagen have been approved by the U.S. Food and Drug Administration. This includes the Infuse Bone Graft and LT-Cage, (Medtronic Sofamor Danek USA, Inc., Memphis, TN, USA) based on a collagen scaffold containing recombinant human BMP2 (rhBMP-2) and a collagen scaffold with metal and rhBMP-2, respectively. Both products are intended for bone void fillers and osteoinduction [64].

### 2.6. Synthetic Biopolymers

Synthetic polymers used for bone regeneration include poly (lactic-co-glycolic acid) (PLGA), poly (ε-caprolactone) (PCL), poly (lactic acid) (PLA), and poly (ethylene glycol) (PEG). Unlike natural biopolymers, modifying the structure of synthetic polymer structures is less difficult [65]. PCL is characterized by high mechanical strength and biocompatibility; however, its hydrophobic nature limits cell adhesion and proliferation [65]. PLGA has been proven to be the most widely used biodegradable polymeric biomaterial. Developments in medicine and technology also enable PLGA to be used as one of the components of modern biocomposites. The combination of PLGA with well-known bioceramics (i.e., HAp, calcium phosphate) and BGs provides a scaffold for the bioengineered replacement of bone defects. These components, placed in the PLGA structure, are involved in the rapid initiation and maintenance of bone remodeling [66,67,68]. 

The limitations of synthetic polymers (i.e., PLGA and PCL), such as reduced osteoconductive properties; insufficient mechanical strength; and inhibition of cell adhesion, proliferation, and tissue inflammation, have been overcome by the formulation of nanocomposites with osteogenic and anti-inflammatory components [65,69]. It has been shown that 3D printed PLGA/β-TCP scaffolds continuously releasing Zn ions promote high osteogenic and anti-inflammatory properties related to the canonical Wnt pathway, P38 MAPK, and NFkB pathways [69] (Table 6).

In an in vitro study, researchers combined PLGA and Cu-loaded, Zn-based ZIF-8 nanoparticles using 3D printing technology. They reported that the scaffold promoted the proliferation of murine MSCs, facilitated cell adhesion and spreading, induced osteoblastic differentiation of MSCs, and showed remarkable antibacterial characteristics [70]. The application of POSS-PCL nanocomposites up-regulated the expression levels of VEGF and Runx2 and did not cause oxidative stress or cytotoxicity [71]. Another approach recently showed that PCL/GO scaffolds with a gelatin coating improved cell proliferation, promoted cell adhesion, and increased viability and biocompatibility effects [72]. To improve the mechanical strength, cellular affinity, and antimicrobial properties of PLA, fabricated electrospun fibers of PLA containing BG-MgONPs were proposed. These fibers increased scaffold stiffness, which enhanced cell growth and increased ALP expression and antimicrobial behavior [73].

## 3. Challenges and Future Directions

The risk of infection, inflammatory response, and insufficient integration into host tissue are the most common factors restricting the use of orthopedic implants. The incorporation of several nanoparticles such as Mg^2+^, chromium oxide (Cr_2_O_3_), and growth factors (such as BMP-2 and TGF-β1) into BNBM composites can overcome these obstacles. For instance, a significant decrease in bacterial growth in *Staphylococcus aureus*, *Pseudomonas aeruginosa,* and *Escherichia coli* strains was observed after culturing in the presence of Mg^2+^ substituted nanostructured HAp. It was also proven that the Mg^2+^/HAp antibacterial effect was associated with the material surface topography and its electroactive behavior [74]. The incorporation of growth factors such as BMP-2 and TGF-β1 into nanocomposite scaffolds demonstrates sustained release kinetics, growth, proliferation, and osteogenic differentiation of the osteoblasts and MSCs [75]. Scaffolds containing Cr_2_O_3_ nanoparticles can provide a protective effect to osteoblasts in oxidative stress conditions by reducing cell cytotoxicity and recovering mineralization [76].

As structures of polysaccharide-based membranes are unstable under wet conditions, the newest studies have been focusing on improving the wet mechanical properties of polysaccharide-based nanocomposites. An interesting approach reports a fabricated membrane assembled by a BC nanofiber network calcium-crosslinked with a hydrated sodium alginate molecule network. A network of molecules makes the membrane more hydrated and flexible. Additionally, the nanofibers provide better strength and resilience. As a result, a heterogeneous crosslink-and-hydration dual-scale network can fabricate damage-tolerant nanocomposites under wet conditions. This may be potentially applied in guided bone regeneration [77].

Alginate nanocomposites can also be combined with diverse nanofillers to improve their mechanical strength, regulate degradation rates, and optimize therapeutic effects. The incorporation of sodium alginate based nanocomposites with metal nanoparticles not only strengthens their mechanical properties but also renders them effective antibacterial and antifungal agents for various biomedical applications [78,79]. SiO_2_-filled alginate–gelatin nanocomposites represent a promising scaffold with structural integrity for cartilage tissue engineering, offering enhanced mechanical strength and stability. These composites degrade at controlled rates while effectively supporting cell viability and attachment [80]. However, considerable challenges, including scaling up production, maintaining uniformity in nanoparticle distribution [81], managing potential immune responses or toxicity from both the alginate matrix and nanoparticles [82], and ensuring reproducibility [83], present significant obstacles to the clinical application of alginate nanocomposites. For instance, a Cu sulfide nanoparticle-incorporated HAp injectable hydrogel up-regulated the expression of VEGF in the wound area, increased collagen deposition, and enhanced angiogenesis [84].

Several prospective BNBM nanocomposites show potential for regenerating bone defects, and their efficacy has been tested under in vivo conditions using animal models. Nanocomposites developed with citric acid and HAp nanocrystals implanted in osteochondral defects in a rabbit model exhibited integration with the bone. Proof-of-concept HAp nanocomposites showed long-term, 26-week biocompatibility and favorable tissue ingrowth [85]. Another study reported in vivo implantation of scaffolds composed of poly (1,8 octanediol-co-citrate), β-TCP, and cerium oxide nanoparticles in a rat model that exhibited biocompatible properties. Importantly, cells were able to infiltrate through the scaffolds. The surrounding tissues exhibited a minimal immune response, and there were signs of scaffold degradation after 30 days of implantation [86]. However, research on the structural integrity, mechanical characteristics, corrosion properties, and potential cytotoxicity is still necessary [86].

Although natural and synthetic biopolymers can effectively act as a bone replacement scaffold, offering bioresorbable, biocompatible, and osteoconductive characteristics, their main drawbacks include poor osteoinductivity, osteointegration, and bioactivity [87]. Incorporating nanoparticles into BNBM composite scaffolds enhances factors and mechanisms involved in bone regeneration processes and prevents inflammation. For example, adding nanoparticles such as AgNPs and MgONPs, or loading various nanoparticles with antibiotics like minocycline or ciprofloxacin, provides antimicrobial properties [40,46,49,63,88,89]. The application of osteogenic factors such as BMPs and dexamethasone into nanocomposites can up-regulate osteogenesis, reduce osteoclast activation, and decrease acute inflammation at the implanted site [40,63].

The mechanical properties of the scaffold must be as similar as possible to the replaced bone tissue to prevent bone loss, osteopenia, or stress shielding. BNBMs have relatively low mechanical strength and susceptibility to degradation, which may limit their use for bone regeneration or replacement. For example, bioceramics and metals exhibit much higher hardness values and abrasion resistance than biopolymers; therefore, they are often preferred over biopolymers due to their superior strength and durability. A scaffold must have enough mechanical strength to retain its structure and follow its mechanical function after its implantation in the case of hard, load-bearing tissues such as bone. To address this challenge a novel processing technique or the incorporation of several nanoparticles to strengthen the material without compromising its porosity is continuously explored by researchers. The functionalization of BNBM composites with nanoparticles can lead to improvements in their mechanical properties, such as strength, flexibility, and durability. For example, several studies reported a successful improvement of the mechanical properties of fabricated alginate scaffolds by incorporating NC, Zn-Sr-BGNPs, or cellulose incorporating MgONPs.

Future directions for bone replacement nanocomposites should involve the development of a novel class of polymers consisting of nanoparticles, such as shape memory polymers (SMPs) and self-healing polymers (SHPs). SHP nanocomposites consist of quadruple hydrogen bonding between composite constituents, which has been shown to enhance self-healing capabilities. These nanocomposites can autonomously and spontaneously recover their properties after damage or fracture, resulting in a wide array of applications not only in bone tissue engineering but also in cardiac valve repair and wound healing [90,91]. SMPs can maintain their shape after the application of a temporarily applied force and withstand vast amounts of pressure. Outside of medical engineering, SMPs also have applications in space structures and textiles. A recent study proved that nano-SiO_2_ can improve the mechanical and shape memory properties of PLA-based copolyester, exhibiting a decent shape fixity and recovery ratio with a trigger temperature that is around body temperature. Due to their low trigger temperature, phase change materials with latent heat energy storage have potential applications in biomedical areas, such as smart punctual plugs [92].

## 4. Conclusions

Recent advancements in the treatment of bone defects utilizing nanocomposites have encountered challenges during in vivo evaluations, resulting in unpredictable clinical outcomes and an inability to effectively restore functional bone tissue affected by diseases and fractures. BNBM nanocomposite scaffolds present significant potential for enhancing bone regeneration techniques, as they can replicate the intricate architecture of natural bone tissue. Preclinical studies, both in vitro and in vivo, have shown that these nanocomposite scaffolds possess the ability to regenerate bone defects concurrently. However, further optimization and refinement of these scaffolds are necessary to successfully transition from laboratory studies to clinical applications. Future investigations should prioritize the development of nanocomposite scaffolds that accurately emulate the extracellular matrix and structure of natural bone while also incorporating antibacterial and anti-inflammatory properties, thereby paving the way for innovative and effective bone treatment strategies with improved clinical results.

## Figures and Tables

**Table 2:** Alginate-based scaffolds, prospects, and limitations.

Nanoparticles	Scaffold Composition	Prospects/Limitations	Ref.
PGO ^1^ and HAp ^2^	PGO ^1^ and HAp ^2^—incorporated on AG ^3^	ROS ^4^-scavenging ability; anti-inflammatory activity; immunomodulatory properties.	[31]
nanoceria, NC ^5^	NC ^5^ incorporated on gelatin–alginate scaffolds	Increased mechanical properties; biomineralization; decreased swelling; improved cell attachment, cell proliferation, and viability; accelerated osteogenic differentiation; reduced free radicals.	[32]
Zn-Sr-BGNPs ^6^	Alginate scaffold with incorporated Zn-Sr-BGNPs ^6^	Improved mechanical performance of the scaffolds; decreased swelling rates; enhanced calcium deposition; increased bone cell proliferation. Further investigation of behaviors, interactions, and responses of cells and the scaffolds is needed.	[33]
PDA/SiO_2_-CaO ^7^	ADA-GEL ^8^ scaffolds incorporating PDA)/SiO_2_-CaO ^7^ modified with BSA ^9^	Improved mechanical stability; elasticity; biodegradation; enhanced bioactivity; cell adhesion; proliferation; higher ALP ^10^ activity. Long-term biocompatibility of 3D printed scaffolds is needed.	[34]
Alg/GO/Ser/nHAp ^11^	Alg/GO/Ser/nHAp ^11^	Desirable mechanical strength; porosity; biocompatibility; osteogenic differentiation. Further investigation of osteoimmunomodulation is needed.	[35]

^1^ Polydopamine-mediated graphene oxide (PGO); ^2^ hydroxyapatite nanoparticles (HAp); ^3^ alginate/gelatin (AG); ^4^ reactive oxygen species (ROS); ^5^ cerium oxide nanoparticles (NC); ^6^ zinc and strontium sol–gel-derived bioactive glass nanoparticles (Zn-Sr-BGNPs); ^7^ polydopamine/silica dioxide-calcium oxide (PDA/SiO_2_-CaO); ^8^ alginate dialdehyde–gelatin (ADA-GEL); ^9^ bovine serum albumins (BSA); ^10^ alkaline phosphatases (ALPs); ^11^ alginate/graphene oxide/sericin nanohydroxyapatite (Alg/GO/Ser/nHAp).

**Table 4:** Hyaluronic acid-based scaffolds, prospects, and limitations.

Nanoparticles	Scaffold Composition	Prospects/Limitations	Ref.
AgNPs ^1^	Hydrogel containing *β*-TCP, HA ^2^, corn silk extract, and nanosilver	Antibacterial activity; lack of cytotoxicity; appropriate biocompatibility; promoting high bone differentiation of MSCs ^3^. Synthesis of AgNPs ^1^ in an aqueous medium of corn silk extract without the use of toxic chemical reagents makes them more suitable for clinical and biomedical applications.	[46]
SLNs ^4^	HA ^2^-PCLA ^5^ hydrogel with SLNs ^4^	Decreasing macrophage polarization; angiogenetic effects; anti-osteoclastic differentiation; promoting defective bone regeneration through immunomodulation. There is a lack of validation in larger animals.	[47]
MBG ^6^ nanoparticles loaded with ipriflavone	MBG ^6^ with ipriflavone incorporated on HA ^2^	No adverse inflammatory effects; mildly enhanced angiogenesis; higher presence of osteoblasts. Further tests need to be performed.	[48]
MBGNs ^7^ loaded with MNCl ^8^	Photo-crosslinkable HA ^2^ and MBGNs ^7^	Inhibition of the expression of pro-inflammatory genes; lack of toxicity; inhibition of the proliferation of *Streptococcus* mutants. The precise time sequences during the treatment, dosage of nanoparticles, and their drug loading areas need to be studied.	[49]

^1^ Silver nanoparticles (AgNPs); ^2^ hyaluronic acid (HA); ^3^ mesenchymal stem cells (MSCs); ^4^ quercetin-solid lipid nanoparticles (SLNs); ^5^ hyaluronic acid -ɛ-caprolactone-co-lactide (HA-PCLA); ^6^ mesoporous bioactive glass (MBG); ^7^ mesoporous bioactive glass nanoparticles (MBGNs); ^8^ minocycline hydrochloride (MNCl).

**Table 5:** Collagen-based scaffolds, prospects, and limitations.

Nanoparticles	Scaffold Composition	Prospects/Limitations	Ref.
Sr-GO ^1^	Sr-GO ^1^ nanocomposites in collagen	Facilitates cell adhesion and osteogenic differentiation.	[60]
nSC ^2^	nSC ^2^	Induce repair of bone defects without the use of exogenous cells and growth factors; activate multiple signaling pathways related to MSC ^3^ recruitment and bone regeneration.	[58]
Strontium and MSCs ^3^	Hydrogel collagen nanocomposite with 2% strontium in the presence of MSCs ^3^	Enhanced osteoblastic production of OCN ^4^ for 28 days.	[61]
SIM ^5^ loaded ACMP ^6^ nanocomposites	ACMP ^6^/SIM ^5^/collagen scaffolds	High cytocompatibility, promotes the proliferation and osteogenic differentiation of pre-osteoblastic cells; upregulation of bone regeneration.	[62]

^1^ Strontium–graphene oxide (Sr-GO); ^2^ nanosilica–collagen scaffolds (nSCs); ^3^ mesenchymal stem cells (MSCs); ^4^ osteocalcin (OCN); ^5^ simvastatin (SIM); ^6^ amorphous calcium magnesium phosphate (ACMP).

**Table 6:** Synthetic biopolymers-based scaffolds, prospects, and limitations.

Nanoparticles	Scaffold Composition	Prospects/Limitations	Ref.
Zn ^1^ submicron particles	PLGA ^2^/β-TCP ^3^/Zn ^1^	Exhibited anti-inflammatory properties; promoting cell adhesion and osteogenic differentiation; improved mechanical properties. The controlled release of Zn ^1^ should keep Zn ^1^ at a low concentration to reduce its toxicity.	[69]
Copper-loaded-ZIF-8 ^4^ nanoparticles	PLGA ^2^/Cu ^5^ (I)@ZIF-8 ^4^	Promote the proliferation of murine MSCs ^6^; induced osteoblastic differentiation of MSCs ^6^; antibacterial characteristics.	[70]
POSS ^7^	POSS ^7^-PCL ^8^ nanofiber	High biocompatibility; lack of cytotoxicity; no oxidative stress.	[71]
Gt ^9^	PCL ^8^/GO ^10^ scaffolds with Gt ^9^ coating	GO ^10^ decreased fiber diameter; increased scaffold stiffness, and improved gelatin coating process; Gt ^9^ coating improved cell proliferation. Evaluation of lower percentages of GO ^10^ and longer culture times should be carried out to assess proliferation.	[72]
n-BG ^11^ and n-MgO ^12^ nanoparticles	PLA ^13^/n-BG ^11^/n-MgO ^12^ scaffolds	Antimicrobial activity of n-MgO ^12^; increased ALP ^14^ expression; promoted cell growth on the surface of the fibers; high osteoblastic phenotype expression capacity of n-BG ^11^.	[73]

^1^ Zinc (Zn) ^2^ poly (lactic-co-glycolic acid) (PLGA); ^3^ β-tricalcium phosphate (β-TCP); ^4^ zeolitic imidazolate framework-8 (ZIF-8); ^5^ copper (Cu); ^6^ mesenchymal stem cells (MSCs); ^7^ polyhedral oligomeric silsesquioxane (POSS); ^8^ polycaprolactone (PCL); ^9^ gelatin (Gt); ^10^ graphene oxide (GO); ^11^ bioactive glass (n-BG); ^12^ magnesium oxide (n-MgO); ^13^ poly (lactic acid) (PLA); ^14^ alkaline phosphatases (ALPs).
